# Guillain-Barré Syndrome after H1N1 Shot in Pregnancy: Maternal and Fetal Care in the Third Trimester—Case Report

**DOI:** 10.1155/2012/323625

**Published:** 2012-12-05

**Authors:** Felipe Favorette Campanharo, Eduardo Felix Martins Santana, Stephanno Gomes Pereira Sarmento, Rosiane Mattar, Sue Yazaki Sun, Antonio Fernandes Moron

**Affiliations:** Department of Obstetrics, Paulista School of Medicine, Federal University of São Paulo (UNIFESP), Alameda das Boninas, 299 Apartamento 112, Vila Mariana, 05303-000 São Paulo, SP, Brazil

## Abstract

Guillain-Barré syndrome is a rare neurological disease of progressive installation, usually following a previous acute infectious state, has a rare incidence, especially in pregnancy, and can induce major complications and high mortality risk. Its occurrence, after immunization to influenza during the last trimester pregnancy, has not been reported before. We presented a case of a 36-year-old pregnant woman that was immunized to H1N1 in the last trimester; 10 days later she developed shoulder and lumbar spine's pain, limbs weakness and facial paralysis with unfavorable clinical evolution and was submitted to intensive therapy care. We described clinical and obstetrical approach, pointing out peculiarities involved in this pathology in pregnancy.

## 1. Introduction

Guillain-Barré syndrome (GBS) is an acute inflammatory polyneuropathy demyelinating of autoimmune etiology with incidence ranging from 0,75 to 2 cases per 100.000 people a year [1]. The disease occurrence in the last trimester of pregnancy increases maternal risks of respiratory complications, needing ventilatory support in 34,5% of the cases and increasing risk of prematurity. Maternal mortality and is reported in 10% of the cases, despite high complexity medical assistance [2].

It begins with ascending and progressive weakness of legs and arms and areflexia, evolving from hours to days, lasting three weeks for the whole installation. Paraesthesias and cranial nerves involvement often happen, and bladder dysfunction is found in most serious cases, usually transitory. 

Around 75% of patients report an acute infection preceding the disease in 1–3 weeks. The GBS association with vaccination to influenza is rare. The incidence is less than a case by 100.000 shots and less than 3% before 20 years old [3].

We report a GBS case after immunization to influenza on pregnancy, in which early diagnosis and therapeutic measures were necessary in order to restore maternal and fetal well being. It presented the importance of clinical propaedeutic and assistance especially in the last trimester of pregnancy and in appropriate management of labor.

## 2. Case Presentation

36-years-old patient, African descendent, gravida 4 para 1, went to São Paulo's Hospital at Paulista School of Medicine (Federal University of São Paulo, UNIFESP), 36-week-gestation, complaining of shoulder and lumbar spine's pain and limbs weakness from the last 20 days. She developed progressive facial paralysis, dysphagia mainly for liquids, dyspnoea, diplopia, and urinary and fecal incontinence. The patient had previously received H1N1 shot 10 days before the onset of symptoms. She was conscious and alert, hemodynamically stable, and with no signs of respiratory distress. 

Neurological evaluation showed diplopia at right, facial diparetic worse at right, motor strength in degree 4 in upper limb and in degree 3 in lower limb, global areflexia, global hypoesthesia especially distal region of hands and feet, and dysarthric speech. Obstetrical examination showed uterine height adequate to pregnancy period, single fetus, alive, and cephalic presentation. Laboratory investigation excluded infection, and cerebrospinal fluid analysis revealed proteins 114 mg/dL, glucose 76 mg/dL, and 6 leukocytes. She was admitted to intensive care unit and immediately received intravenous immunoglobulin 2 g/kg during the following 5 days, besides obstetrical, psychotherapy, physiotherapy, and speech therapy support.

After the first day, there was a clear improvement in dysphagia and of proximal limb strength. On the 4th day the patient was discharged from ICU and received in the obstetric ward to undergo support and finish the treatment. 

All fetal parameters checked (ultrasonography and cardiotocography) were normal throughout the period of hospitalization. Carbamazepine prescription was used for neuropathic pain. She was discharged on the 14th day, forwarded to risk prenatal care at UNIFESP. On the 21st day, 38-week-gestation was admitted to child-birth unity for spontaneous labor, with uneventful vaginal birth, giving birth to a 2640 g male newborn, Apgar 9 in the first minute and 10 in the fifth minute. No anesthetic procedure was performed. Early postpartum period had good evolution, and only low urinary incontinence was observed, being referred for pelvic floor physiotherapy.

## 3. Discussion

Guillain-Barré syndrome is a rare and dangerous neurological disease especially during pregnancy. Its occurrence can be established in 13% in the first trimester, 47% in the second trimester, and 40% in the third trimester [4]. The initial report can be similar to many neurological diseases and often confused with pregnancy symptoms, and the clinical exam is an important tool to diagnose specially if it is accompanied by albuminocytological dissociation in cerebrospinal fluid, that it is a common finding in most cases [5].

Controlled and randomized studies showed plasmapheresis effectiveness in the GBS treatment; however, its use was related, among other complications, with potential risk of hypotension and septicemia [6]. Intravenous immunoglobulin is used since the 80's decade and, despite having similar efficacy to plasma exchange, is preferable because of the advantage of bringing lower risk of complications, besides presenting evidence level A for treating this disease [7, 8]. Carbamazepine is effective in controlling neuropathic pain associated with GBS. However, its use should be avoided in the first trimester of pregnancy by the teratogenic potential [9].

Anesthetic procedures are at risk for these patients. General anesthesia carries significant risk of hyperkalemia associated with the use of succinylcholine, and frequent autonomic instability easily was found in these cases. Regional blockage seems to worsen hypoesthesia and neurological syndromes [4].

In this case, an appropriate management of the patient was performed, allowing fast reversal of the situation and favorable outcome of pregnancy. This is the first case of Guillain-Barré syndrome after immunization to H1N1 in the third trimester of pregnancy.

Early GBS diagnosis must be done in order to achieve an effective treatment and rather under multidisciplinary care. Prevention of adverse events, proper management of pain, and control of the patient's anxiety are fundamental, even more critical in pregnancy and puerperium.

In conclusion, people should clearly understand that the risk of being infected by Guillain-Barré syndrome is much less than the chance of getting influenza, being hospitalized, and dying from flu complications. The vaccine remains effective and necessary for successful public health program.
